# From a consortium sequence to a unified sequence: the *Bacillus subtilis* 168 reference genome a decade later

**DOI:** 10.1099/mic.0.027839-0

**Published:** 2009-06

**Authors:** Valérie Barbe, Stéphane Cruveiller, Frank Kunst, Patricia Lenoble, Guillaume Meurice, Agnieszka Sekowska, David Vallenet, Tingzhang Wang, Ivan Moszer, Claudine Médigue, Antoine Danchin

**Affiliations:** 1 CEA, Institut de Génomique, Génoscope, 2 rue Gaston Crémieux, 91057 Évry, France; 2 CEA, Institut de Génomique, Laboratoire de Génomique Comparative/CNRS UMR8030, Génoscope, 2 rue Gaston Crémieux, 91057 Évry, France; 3 Institut Pasteur, Intégration et Analyse Génomiques, 28 rue du Docteur Roux, 75724 Paris Cedex 15, France; 4 Institut Pasteur, Génétique des Génomes Bactériens/CNRS URA2171, 28 rue du Docteur Roux, 75724 Paris Cedex 15, France

## Abstract

Comparative genomics is the cornerstone of identification of gene functions. The immense number of living organisms precludes experimental identification of functions except in a handful of model organisms. The bacterial domain is split into large branches, among which the Firmicutes occupy a considerable space. *Bacillus subtilis* has been the model of Firmicutes for decades and its genome has been a reference for more than 10 years. Sequencing the genome involved more than 30 laboratories, with different expertises, in a attempt to make the most of the experimental information that could be associated with the sequence. This had the expected drawback that the sequencing expertise was quite varied among the groups involved, especially at a time when sequencing genomes was extremely hard work. The recent development of very efficient, fast and accurate sequencing techniques, in parallel with the development of high-level annotation platforms, motivated the present resequencing work. The updated sequence has been reannotated in agreement with the UniProt protein knowledge base, keeping in perspective the split between the paleome (genes necessary for sustaining and perpetuating life) and the cenome (genes required for occupation of a niche, suggesting here that *B. subtilis* is an epiphyte). This should permit investigators to make reliable inferences to prepare validation experiments in a variety of domains of bacterial growth and development as well as build up accurate phylogenies.

## INTRODUCTION

*Bacillus subtilis* has been a model for Gram-positive bacteria for more than a century. Generally Recognized As Safe (GRAS), it is a ubiquitous ingredient of food supplies and a typical member of the A+T-rich Firmicutes, a major clade of the bacterial domain of life. It has been used as the reference model for cell differentiation, and many studies have analysed its well-defined sporulation programme (for reviews see Errington, 2003; Piggot & Hilbert, 2004; Yudkin & Clarkson, 2005). Whereas it has long been accepted that model organisms play a crucial role in the way they coordinate the research of many investigators, some now minimize the input of laboratory organisms that, historically, have been chosen in a more or less random way (Hobman *et al.*, 2007). Yet, we need references, and comparative genomics is based on approaches that have much in common with the way hieroglyphics were understood using the Rosetta stone. This was an obvious reason for the setting up of a *B. subtilis* genome sequencing programme in 1987. At that early time of genomics, cloning pieces covering the entire genome as well as sequencing was a tedious task. Furthermore, it was obvious, if one wished to couple sequencing to functional knowledge, that the various expertises of many investigators should be put together in one common programme. This was at the core of the European effort to set up two major consortia for sequencing microbial genomes, the *Saccharomyces cerevisiae* consortium and the *B. subtilis* consortium (Simpson, 2001). Joined by Japanese investigators in 1990, the consortium extended to some 30 groups (including two US and a Korean group, joining later on) which sequenced and annotated chromosomal segments covering the whole *B. subtilis* genome (Harwood & Wipat, 1996).

Whereas this mode of organization had obvious benefits in terms of creation of scientific knowledge, it had technical drawbacks. Indeed, putting together different laboratories implied different local practices and different performances in the quality of the final sequence. Furthermore, because of the difficulty in sequencing at that early time, some regions which had been sequenced in previous years were not resequenced (see Results and Discussion). Finally, the sequencing techniques were quite time-consuming and involved cloning into a variety of vectors, which could result in alteration of the original sequence, especially given that *B. subtilis* DNA is often toxic in *Escherichia coli* (Frangeul *et al.*, 1999). Being aware of this problem, the *B. subtilis* consortium decided to use a first final draft of the sequence to identify regions potentially containing inaccuracies, to PCR out and resequence 500 bp fragments in these regions (Kunst *et al.*, 1997; Medigue *et al.*, 1999). However, there was no doubt that errors still remained in the published sequence. Naturally, sequencing was the prelude to an explosion of new genetic, physiological and biochemical analyses, and genes which were initially of unknown function were constantly being discovered. The first reference database, SubtiList, displaying sequence and annotations, was replaced six years ago by an update (Moszer *et al.*, 2002). However, no further recent updates of the sequence and the annotation repository are available and this should become a matter of concern as *B. subtilis* remains widely used in automatic annotation procedures.

The 1997 sequence was used as a reference until the present time. Since then sequencing techniques have improved dramatically. They no longer require cloning steps, which, in the case of A+T-rich Firmicutes (excluding Mollicutes, which do not express well most of their DNA in *E. coli* because of their use of a UGA codon encoding tryptophan), counterselect regions that are expressed at high levels in the library hosts (Frangeul *et al.*, 1999). Furthermore, it has been repeatedly observed that bacterial strains evolve fast in laboratories. For example *E. coli* strains MG1655 and W3110 are significantly different (including a large inversion around the origin of replication) (Hayashi *et al.*, 2006; Herring & Palsson, 2007). It has also been found that isolates of MG1655 from different laboratories may display considerable variations (Soupene *et al.*, 2003). These observations should be extended to *B. subtilis*, and it was therefore timely to resequence the genome to have a reliable reference.

Per se a genome sequence is of limited interest. What is important for the scientific community is the identification of the genomic objects present in the sequence, associated with their functions experimentally identified or predicted *in silico*. In fact, while we do not have exact measures of the impact of annotations, we know how errors percolate in a very dangerous process (Gilks *et al.*, 2002). It is interesting to note that many genes and gene products retained the ‘y’ name created during the first sequencing programmes, testifying that the genome project has indeed been seminal to many discoveries, even if no reference to the original annotation work was provided. We therefore decided to reannotate the sequence entirely using a recently developed platform, MaGe [Magnifying Genomes (Vallenet *et al.*, 2006)], systematically proceeding by inference and using all kinds of neighbourhoods (including *in biblio*, with the systematic use of selected PubMed references, and using PubMed Central as much as possible), as described previously (Nitschke *et al.*, 1998). To keep in phase with the international community, a jamboree was organized by the Swiss Institute of Bioinformatics in Geneva, to harmonize our annotation with that of the HAMAP project (Lima *et al.*, 2009).

The knowledge of the genome sequence allows one to explore the consistency of annotations and, in particular, the organization of the genome into several distinct functional processes: what sustains life, what perpetuates life, and what permits life in a particular niche (Danchin *et al.*, 2007). The latter is an essential motivation for the choice of an organism as a useful model. Does analysis of the *B. subtilis* genome fit with its biotope? Back in 1859, Louis Pasteur thought he had clearly demonstrated the absence of spontaneous generation in broths, usually found to become rapidly full of living microbes. His (in)famous competitor Félix Pouchet fought back heartily, showing that he found evidence contrary to that of Pasteur, and a bitter fight ensued. What was Pouchet's evidence? Pasteur used heated yeast extracts while Pouchet showed that he recovered micro-organisms after having boiled vessels using ‘l'eau de foin’ (hay water) (Roll-Hansen, 1979). We now know the reason why this is so: hay is the normal niche of a bacterium identified in 1885 as the ‘hay bacterium’, *B. subtilis*, which makes heat-resistant spores, and is found as a major component in the process of water retting of plants used, in particular, for the production of linen threads (Tamburini *et al.*, 2003).

In this work we used the complete resequencing of the genome to emphasize some new features of the genome, the genes and their products, leaving more standard information readily available in two databases: a new updated release of SubtiList, now integrated in a multi-genome framework, GenoList, and BacilluScope, the overview of the annotation platform MaGe.

## METHODS

### Origin of strain and DNA.

The *Bacillus subtilis* strain used in this work is the one described by Anagnostopoulos and Spizizen (Anagnostopoulos & Spizizen, 1961; Zeigler *et al.*, 2008). It is the same as the one which was distributed to the consortium involved in the sequencing of strain 168, beginning in 1987 (Kunst *et al.*, 1997). Many strains labelled ‘168’ exist in many laboratories in the world, and it is expected that there is significant polymorphism in these strains. Indeed, recent work has identified variations, some of which differ both from the published sequence and from the update presented in this work (Srivatsan *et al.*, 2008).

*B. subtilis* strain 168 had been distributed to the sequencing consortium initially in 1989–1990. However, it has already been noticed that after 20 years of passages in many laboratories the growth phenotype of the strain differed from place to place. In particular this was evidenced by variations in the growth rate on minimal media supplemented with ammonium as a nitrogen source (E. Presecan & A. Sekowska, unpublished observations; see Results and Discussion). For this reason a new culture derived from the original collection of *B. subtilis* strain 168 conserved by C. Anagnostopoulos was used for the functional analysis programme (Kobayashi *et al.*, 2003). The genome of this same isolate has been sequenced in the present work.

*B. subtilis* chromosomal DNA was purified as described by Saunders *et al.* (1984).

### Sequencing data: directed vs *de novo* sequence assembly.

Two strategies might have been used to assemble the *B. subtilis* 168 genome sequence. On the one hand, the assembly could have been achieved via a directed assembly procedure which would have used the original sequence of strain 168 as a template to orient and organize the contigs produced by the assembler software. However, this approach, which *de facto* presumes collinearity between the reference sequence and the newly sequenced material, would have certainly prevented the uncovering of large genomic rearrangements, if any. On the other hand, during a *de novo* assembly procedure, contigs are assembled but their respective orientation and organization cannot be determined, unless paired-end reads are used. Although in this case no assumption is made for the organization of the scaffold, this may lead to the construction of chimaeric contigs. Even though both problem types can be solved easily via PCR experiments, problematic areas on the scaffold had to be pinpointed first. We thus processed the sequencing data as follows.

A total of 346 189 (average length 230 nt) valid single reads were produced using Roche/454-GSFLX technology (Margulies *et al.*, 2005) representing approximately 19× coverage of the final molecule. *De novo* assembly was then performed with the Newbler 2.0 software, leading to 51 contigs (larger than 500 nt) for a total length of approximately 4.2 Mb. *De novo* assembled contigs were remapped using the Mummer3 software (Kurtz *et al.*, 2004) onto the reference sequence and no rearrangement or chimaera cases were revealed since all of them aligned perfectly with the original strain 168 sequence. A further stack of 1542 reads were generated by sequencing PCR products to validate the contig organization and orientation as well as to fill potential remaining sequence holes which did not encompass the location of rDNA clusters.

As they correspond to repetitive regions, rDNA clusters were logically discarded during the assembly procedure and their sequences were determined using a sequencing technology which generates longer fragments than 454-GSFLX. All but one rDNA cluster were successfully resequenced using Sanger technology. The large cluster comprising the three rDNA *H*, *G* and *I* clusters could not be properly resolved (i.e. one rDNA cluster was found instead of three). This raised two hypotheses: (i) a significant genomic rearrangement occurred during *B. subtilis* 168 evolution (e.g. recombination between clusters *I* and *G*) or (ii) PCR could not be successfully performed due to particular DNA secondary structures in this region (stem–loop structures). However, since spacers between rDNA subunits often contain tRNAs, it was possible to validate or invalidate one of the hypotheses by searching in sequence reads for tandems or, if any, triads of tRNAs that are characteristic of the large cluster *rrnIHG* (see Supplementary Table S1, available with the online version of this paper). One such triad theoretically exists (tRNA*arg*–tRNA*gly–*tRNA*thr*), but its length (370 bp) greatly exceeds the average length of reads produced by pyrosequencing in the present work. Luckily, tandems tRNA*arg*–tRNA*gly* and tRNA*gly–*tRNA*thr*, which are still specific to the *rrnIHG* cluster, were found in 42 reads out of 305 containing two complete tRNAs. This strongly suggested that PCR experiments failed for that particular region; hence we incorporated the original sequence of *rrnIHG* in our final assembly.

### Consensus sequence correction.

To identify possible sequencing errors, a script based on the ssaha2 aligner (Ning *et al.*, 2001) was written. This script takes as input the reference molecule as well as the sequencing data (i.e. sequence reads and associated base quality), remaps reads onto the reference sequence and reports a list of potential variations between this reference sequence and the newly sequenced material. The events reported can be either simple substitution or insertion or deletion events. This analysis led to the detection of more than 2000 potential differences (1589 substitutions and 578 indels) between the two *B. subtilis* genomes. All the positions spotted by this approach were manually checked using the consed software (Gordon *et al.*, 1998) and a large proportion of them turned out to be true variations.

However, it is now well known that pyrosequencing technology has trouble in correctly resolving homopolymer runs (Huse *et al.*, 2007). For this reason, we performed two further Solexa runs (Bentley, 2006), which produced 3 214 055 single reads for a 27× coverage of the molecule. Using an alignment process similar to that used for GSFLX reads, 42 positions only could be corrected. This indicated that GSFLX performed remarkably correctly and that the remaining variations were likely to be true.

### Annotation procedures.

Gene prediction was performed using the AMIGene software (Bocs *et al.*, 2003), using the gene models built with the published version of *B. subtilis* annotations. The predicted coding sequences (CDSs) were assigned a ‘locus_tag’ similar to that of the previous *B. subtilis* annotations, i.e. ‘BSUxxxxx’: the CDS number remained the same for identical genes, even in the case of variation in the total length of the alignment between the old and new versions of a gene (corresponding CDS shorter or longer). In the case of additional gene predictions and of fissions or fusions, we used the remaining numbers (1 to 9, as the *B. subtilis* CDSs were numbered from 0 to 90 by tens). For example, the gene fission BSU33220 is now replaced by two new locus_tags: BSU33221+BSU33222. The fusion observed with BSU01840+BSU01850 now corresponds to the CDS BSU01845 (see Supplementary Table S2). The sets of predicted genes were submitted to automatic functional annotation, as described previously (Vallenet *et al.*, 2006). The initial functional assignation was based on the transfer of the *B. subtilis* annotations (Moszer *et al.*, 2002) between gene products presumably differing as the result of sequencing errors, i.e. 85 % identity on at least 80 % of the length of the smallest protein. Sequence data for comparative analyses were obtained from the NCBI database (RefSeq section, http://www.ncbi.nlm.nih.gov/RefSeq). Putative orthologues and synteny groups (i.e. conservation of the chromosomal colocalization between pairs of orthologous genes from different genomes) were computed between the newly sequenced genome and all the other complete genomes as described previously (Vallenet *et al.*, 2006). Each CDS was individually analysed by searching for its name (and the name of synonyms) in the PubMed and PubMed Central data libraries, as well as in Google. PubMed identifiers (PMIDs) were included in the MaGe platform as an information validating the status of the annotation, with particular emphasis on experimental validation of activity. All these data (syntactic and functional annotations, and results of comparative analysis) were stored in a relational database, called BacilluScope. A complete manual validation of the automatic annotation by several users in different locations was performed using the MaGe web-based interface.

### Finding regions of genomic plasticity.

Potential genomic islands in the *B. subtilis* chromosome were searched for with the RGP (region of genomic plasticity) tool implemented in MaGe (Vallenet *et al.*, 2006), which is based on the synteny breaks between genomes compared in parallel (here *B. subtilis*, *Bacillus amyloliquefaciens* FZB42, *Bacillus licheniformis* ATCC 14580 and *Bacillus pumilus* SAFR-032). The predicted regions were checked manually and compared to the chromosomal regions of potentially foreign origin previously detected using hidden Markov models (Nicolas *et al.*, 2002) and Multiple PairWise Test (Merkl, 2004) (Supplementary Table S3). It appeared that these genomic regions sometimes had a composite structure, e.g. they were made of regions partially conserved or found in different synteny groups (i.e. in different genomic locations) in the different *Bacillus* genomes. The predicted RGPs have therefore been further manually curated to define subregions called modules.

### Metabolic reconstruction.

Based on the new functional annotations of the *B. subtilis* genome, the reconstruction of metabolic pathways at work in this bacterium was performed using the Pathway Tools software suite (Paley & Karp, 2006). We first built a BioCyc Pathway/Genome database, which linked annotation data to MetaCyc data, a set of canonical metabolic pathways (Caspi *et al.*, 2008). Then a number of specific sections of the metabolism went through manual curation (see Results and Discussion). Data compiled in the framework of an independent metabolism modelling project were also used, in collaboration with their authors (Goelzer *et al.*, 2008). Altogether, the SubtiliCyc database produced in this way comprises approximately 280 metabolic pathways (both curated and automatically inferred) and 1300 reactions.

## RESULTS AND DISCUSSION

### Distribution of variants between the present sequence and the consortium sequence

**Fig. 1. f1:**
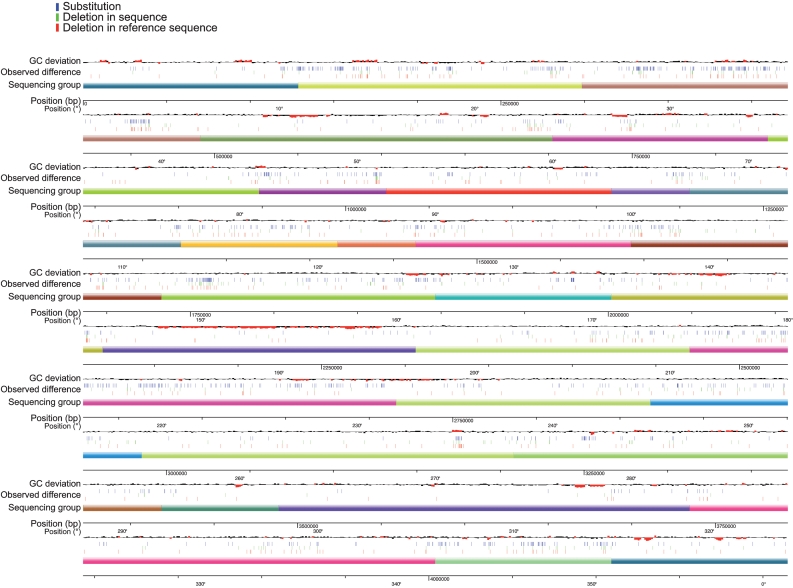
Comparison between the previously published sequence of strain 168 and the strain resequenced without cloning. SNPs and indels are as indicated, as well as the uneven distribution of G+C nucleotides in the sequence. Under the line representing the genome are displayed the positions of the different regions attributed to the various members of the sequencing consortium. It can be seen that the amount of variation is dependent on the sequencing group, not on the nucleotide composition of the genome. In some regions there is precious little variation, compared with the present sequence, despite the fact that the techniques used between 10 and 20 years ago were very different from those used today.

Fig. 1 summarizes the distribution of single nucleotide polymorphisms (SNPs) and other variants along the reference sequence together with coloured boxes spanning the regions that have been assigned to the different groups of the consortium. There is clearly a highly non-random distribution of variations, which correlates with the different regions corresponding to different groups. First, we noticed several regions with only very few differences from the present sequence, and these are essentially very small variations (∼0.860–0.950 Mb; 2140–2310 Mb; ∼3480–3710 Mb). Comparing with variations in other related genomes, or in regions independently sequenced by various groups in the course of their own specific work on a gene or a group of genes, these variations appear most often not to be SNPs, but sequencing errors (see below). Some regions are heterogeneous, with subregions devoid of errors and subregions with a substantial amount of variations: typically this fits well with the way the sequencing programme has been organized at its onset in some laboratories, with pieces of 15–20 kb sequenced by students, who were obviously not all able to ensure the same level of accuracy (Glaser *et al.*, 1993). We also noted that some regions, which have not been resequenced by the consortium but taken as published at the time, carry a higher level of errors (this is the case for ribosomal protein operons; for example see the region around 0.120 Mb, where there is a concentration of variations, while the upstream region is almost without errors).

*B. subtilis* 168 is a laboratory strain which is highly competent for DNA uptake. Its popularity originates from the ease with which it can be genetically manipulated; the exact origin of its increased competence is not well established, as it may well have derived from some physiological change when the wild-type Marburg parent strain was domesticated at Yale University (for reviews, see Earl *et al.*, 2008; Zeigler *et al.*, 2008). This strain and its derivatives have been further subjected to extensive mutagenesis by irradiation or chemical mutagenesis (e.g. using nitrosoguanidine or ethyl methanesulfonate) to obtain a large number of genetic markers required for the establishment of a genetic map. Many additional mutants of *B. subtilis* 168 were also constructed and exchanged between laboratories for other research purposes, such as the study of different cell processes, including sporulation, competence, cell division, metabolism, motility, chemotaxis, swarming, etc. The overall result is that most laboratory derivatives of *B. subtilis* 168 contain chromosomal segments that may differ from the ancestral parental clone of strain 168 whose genome has been sequenced. This may account for some of the differences between the initial (uncorrected) sequence and the revised sequence presented here. However, comparison with the many genomes of Firmicutes we now possess, permitting comparison of conserved regions in proteins, strongly argues against polymorphism generated in the various laboratories of the consortium, and supports the idea that most if not all variations between the published sequence and the present one are sequencing errors (some of which derived from cloning artefacts). Our present work is therefore an update of the sequence annotation of *B. subtilis* strain 168.

### Global features of the genome and the proteome

Genome programmes use the established genome sequence to list the major genomic objects, protein and RNA-coding genes, together with general features, including functional annotation. To this standard list (available in BacilluScope and in GenoList) we have now added new features such as riboswitches, small regulatory RNAs [srRNAs, including a series of seven srRNAs recently identified (Saito *et al.*, 2009)] as well as more elaborate features: genomic islands, local codon usage biases, protein amino acid composition and distribution along the chromosome.

Overall the systematic reassessment of syntactic gene locations and the integration of sequence corrections led to many feature updates, summarized in Table 1. A detailed listing of modified genes is given in Supplementary Table S2. A fairly large number of new, and essentially small genes were annotated (171), amongst them approximately 30 % of pseudogenes or gene remnants, and a few of them resulting from fusions or fissions of previously existing genes. Several sequence corrections corresponded to compensating frameshifts, leaving gene boundaries untouched but generating new and better-conserved internal amino acid motifs. Being short, and resulting in an apparently unbroken open reading frame, errors of this type could not have been detected by the procedure meant to identify possible regions in error (Medigue *et al.*, 1999).

**Fig. 2. f2:**
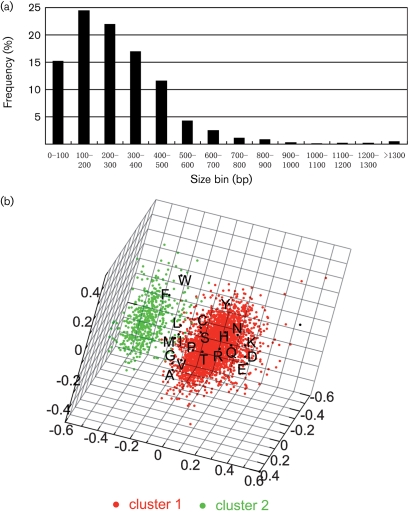
(a) Distribution of gene length in the *B. subtilis* 168 genome. The absence of any overrepresentation of short CDSs supports the view that most if not all gene sequences predicted in the present annotation are authentic. (b) Correspondence analysis of the proteome of *B. subtilis*. Proteins in the proteome can be separated into two well-identified classes. The green cloud corresponds to proteins that are integral inner-membrane proteins (IIMPs). Note that the IIMP cloud is driven by the opposition between charged amino acids (D, E and K) and hydrophobic ones (F, L, M, W).

Correction of errors and manual annotation of small CDSs was analysed by investigating the gene size distribution in the genome (Fig. 2a[Fig f2]). The distribution is quite even, and does not suggest an abnormal distribution of a particular class of lengths, which is the hallmark of spurious CDS identification (Yamazaki *et al.*, 2006).

The relatively small number of error corrections in the present sequence (∼2000, see Methods), relative to the length of the genome implies that the overall analysis of words in the genome does not significantly deviate from our previous studies. In particular, *B. subtilis* is remarkably poor in repeats longer than 25 nt (Rocha *et al.*, 1998, 1999a). As in other bacterial genomes, the constraints placed on the processes of translation initiation and termination result in local compositional biases (Rocha *et al.*, 1999b). Using systematic comparison with CDSs extracted from other genomes (Firmicutes, and *Bacillus* species most often), as well as prediction of exported protein signals, if necessary, we tried to reassign translation start sites in all CDSs. Most are unambiguous, with an excellent ribosome-binding site (RBS, variation on AAGGAGGT) located 4–13 nt upstream of the ATG start codon. In some cases, it is difficult to be absolutely sure of the correct site. In a few cases (*infB*, *lysC*, *pgsB*), a given open reading frame harbours two or more authentic CDSs. This fact needs to be remembered when typical RBSs with a correct start site are found within a long CDS, as other similar cases might have been overlooked.

**Fig. 3. f3:**
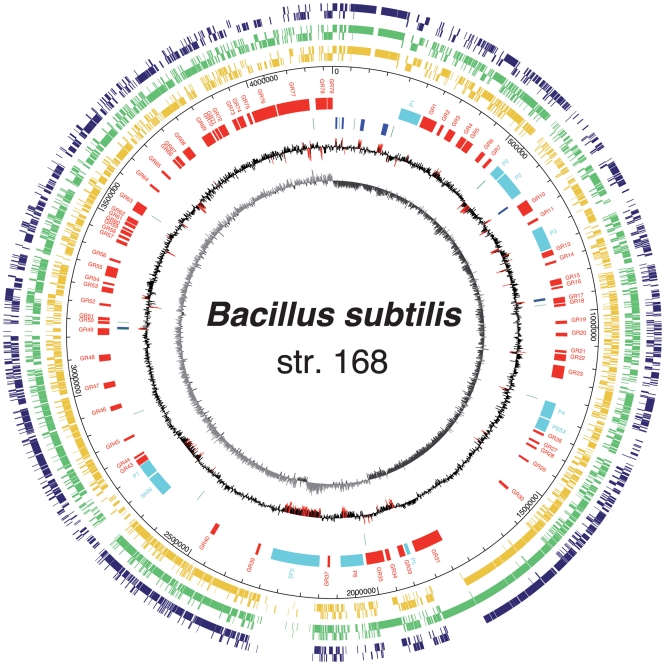
Circular representation of the *B. subtilis* 168 genome for several specific genome features. Circles display the following, from the inside out. (1) GC skew (G+C/G−C using a 1 kb sliding window). (2) GC deviation (mean GC content in a 1 kb window − overall mean GC). Red areas indicate that deviation is higher than 1.5 standard deviation. (3) tRNA (dark green) and rDNA (blue). (4) Location of genomic regions with specific features differentiating them from the average sequence. Boxes coloured in light blue indicate regions of phage origin. The nonsymmetrical distribution (right and left halves of the circle) is to be emphasized. (5) Scale. (6, 7, 8) Genes having a presumed orthologue in other *Bacillus* species (*B. licheniformis*, *B. amyloliquefaciens* and *B. pumilus* respectively).

Our previous work had shown that the genome is heterogeneous when considering many features such as base composition, codon usage biases or functional consistency, with up to ten islands ascribed to prophages or prophage remnants (Bailly-Bechet *et al.*, 2006; Kunst *et al.*, 1997; Moszer *et al.*, 1999). Using the ‘RGP-Finder’ module in the MaGe annotation platform, which allows one to search for regions of genomic plasticity in bacterial genomes, we compared *B. subtilis* with *B. amyloliquefaciens* FZB42, *B. licheniformis* ATCC 14580 and *B. pumilus* SAFR-032. Eighty genomic regions (RGPs) ranging from 5171 nt to 141 234 nt were identified in *B. subtilis* 168 (Supplementary Table S3). Among those, 22 regions covered or overlapped with islands predicted using hidden Markov models (Nicolas *et al.*, 2002) and Multiple PairWise test (Merkl, 2004). These sequences include all of the 10 well-documented genomic regions of phage origin (Kunst *et al.*, 1997), of which only three are integrated at a tRNA gene location and/or contain mobility genes. However, apart from the PBSX phage and region P4, these prophagic regions harbour a significant GC deviation (Fig. 3). Six other predicted RGPs contain at least two specific genomic island features [tRNAs, integrases, mobility-associated genes and pseudogenes (Dobrindt *et al.*, 2004)] and can be named GI-like (genomic island-like). This is the case for GR17, which is made up of two modules (see Methods), one completely specific to *B. subtilis* (three genes: sporulation control gene and two hypothetical proteins), and one absent in *B. pumilus* only (several genes involved in sulfur transport and metabolism). The rest of the predicted RGPs were found using the synteny break point criteria only (Supplementary Table S3), and most often these regions harbour genes coding for enzymic activities and/or transporters. For example, GR48 is made of two modules distinct in terms of functional role: the first one is involved in rhamnogalacturonan transport and utilization, and it is absent in *B. amyloliquefaciens*; the second one contains genes coding for biotin synthase and lysine-8-amino-7-oxononanoate aminotransferase, and it is absent in *B. pumilus.*

Using correspondence analysis we have reinvestigated the distribution of amino acids in the proteome. As reported previously (Pascal *et al.*, 2005), this allowed us to create a list of plausible integral inner-membrane proteins (IIMPs, Fig. 2b[Fig f2] and Supplementary Table S4). We also annotated exported proteins with signal peptides and lipoprotein signal peptides.

### An overview of the cell's organization

Three major processes permit the development of life: sustaining life while combating ageing, propagating life, and living in a particular environment. The first two processes require presumably ubiquitous functions. The third one corresponds to large pools of horizontally transferred genes that are shared by the individual strains of a given species. Ubiquitous functions cannot be derived in any straightforward way from genome comparisons, as they often result from dissimilar structures recruited in the course of evolution. However, the structure of the descent of living organisms implies that there is a tendency for a gene coding for a particular function to be conserved over generations, leading not to ubiquity but to significant persistence. Comparative genomics of bacterial genomes identified a set of persistent genes in two major bacterial clades, the gamma-Proteobacteria (with *E. coli* as the model) and the Firmicutes (with *B. subtilis* as the model organism) (Fang *et al.*, 2005). Detailed analysis of conservation of proximity of genes in genomes showed that both persistent genes and rare genes tend to stay clustered together, making two highly consistent families of genes, separated by a large twilight zone (Danchin *et al.*, 2007).

Remarkably, the connection network of the persistent genes coding for ubiquitous functions is reminiscent of a scenario of the origin of life, forming the *paleome* (from *παλα*ιος, ancient). Two further splits must be made among the functions of the paleome. Some are essential for permitting formation of a colony on plates supplemented by rich medium (Kobayashi *et al.*, 2003); some, while coded by persistent genes, do not have this property (Fang *et al.*, 2005). This particular feature has to be superimposed on a third split, which separates reproduction from replication (Dyson, 1985). Taken together, this view of the paleome opens a novel way to consider genomes and evolution, where management of the creation of information is the central issue when a young organism is born from an aged one. Repeated invention of energy-dependent processes required to make room while accumulating information in a ratchet-like manner probably accounts for the remarkable diversity of the structures involved in the process (Danchin, 2008). The organization of the paleome makes a separation between the machine (which is compartmentalized and sustains metabolism), and the program (which replicates and is expressed both constitutively and under specific conditions).

Finally, bacteria need not only to survive and to perpetuate life, but also to occupy a particular niche. This capability corresponds to a very large class of genes, forming the *cenome* [after *κ*οινος, common, as in biocenose (Danchin *et al.*, 2007)].

We therefore explored the *B. subtilis* genome sequence considering first the genes involved in making, maintaining and repairing the cell, the paleome; and then its cenome, allowing the cell to occupy a specific niche, which defines the features of the organism's biotope as well as those used in industrial applications for instance.

### Compartmentalization

#### The cell membrane.

Phospholipid synthesis and turnover is managed by a variety of processes, encoded by genes *cdsA*, *des*, *dgkA*, *fapR*, *glpQ*, *gpsA*, *lipC*, *mprF*, *pgsA*, *phoH*, *plsC*, *tagA*, *yodM*, *ytlR* and *ytpA*, some of which are essential (Kobayashi *et al.*, 2003). The important process of distribution of phospholipids in the outer layer of the membrane is performed by flippases, which have been at least partially characterized (EpsK, SpoVB, YgaD, YwjA).

#### The cell wall and the cell shape.

The shape of the cell is determined by a variety of processes, combining an internal cytoskeleton coded in particular by *mre*-related genes (for a recent review see den Blaauwen *et al.*, 2008) with synthesis of the murein sacculus (Hayhurst *et al.*, 2008) and its associated teichoic acids (Formstone *et al.*, 2008). The *fts mur* islands are organized in a way compatible with the overall bacillus shape of the organism, as remarked by Tamames *et al.* (2001) in an attempt to correlate the architecture of the cell with the organization of the genome (Danchin, 2009b).

#### Transport.

The genome of *B. subtilis* includes four major classes of transporters: ABC-transporters driven by ATP hydrolysis, phospho*enol*pyruvate-dependent transport systems (PTSs), electrochemically driven permeases (importer and antiporter involving charged substrates) and facilitators. While some have been experimentally analysed [in particular all those related to sucrose transport (Fouet *et al.*, 1987)] many have been annotated by inference (Saier *et al.*, 2002). It should be stressed here that the utmost caution should be exerted when using purely *in silico* analyses in this domain, as it is quite difficult to distinguish related metabolite transporters, including ions. Furthermore there is sometimes fairly wide specificity in the nature of the transported metabolites [e.g. the genes named *tcyJKLM* at the present time transport substrates considerably deviating from cysteine alone (Burguiere *et al.*, 2004; Sekowska *et al.*, 2001)].

### Information transfer

#### Replication, recombination and repair.

Split into three phases, initiation, elongation and termination, these processes are among the best-studied features of the organism (Frenkiel-Krispin & Minsky, 2006; Noirot-Gros *et al.*, 2002) and we did not expect to find much new information from sequence annotation. There is a strong bias of gene expression in the leading strand, and this correlates not only with the presence of two DNA polymerases (DnaE and PolC) as already noticed (Rocha, 2002), but also with that of a series of genes, some of which are of unknown function. Besides this remarkable fusion of two DNA replication apparatuses apparently coming from different origins, an interesting observation was that we repeatedly found association between part of sulfur metabolism and RNA (DNA) degradation in sets of genes that are often associated with poorly understood functions. This substantiates the observation that nanoRNase NrnA (YtqI) is also a 3′-phosphatase that hydrolyses 3′,5′-adenosine bisphosphate, a product of sulfur assimilation, into 5′-AMP (Mechold *et al.*, 2007).

#### Transcription and translation.

Both these processes also follow the standard course: initiation, elongation and termination. Most steps involved in transcription in *B. subtilis* have been analysed for a long time, in particular transcription initiation (Haldenwang, 1995; Kazmierczak *et al.*, 2005; Kunst *et al.*, 1997). We wish however to emphasize the coupling between transcription and DNA repair, which probably needs to be explored much further than the identification of Mfd, the transcription repair coupling factor.

Much progress has been made at the level of translation. Beside a sequence error in protein S12 of the ribosome, previously identified (Carr *et al.*, 2006), a feature of the ribosome is worth noticing. Indeed, several genes code for homologues of previously identified ribosomal proteins, suggesting either involvement during maturation of the ribosome, or involvement in spore ribosomes: *rpmGA*/*rpmGB* (L33), *rplGA*/*rplGB*(*ybxF*) (L7A), *rpsNA*/*rpsNB* (S14) and *rpmEA*/*rpmEB* (L31). A newly identified ribosomal protein, somewhat similar to protein L14E in Archaea, and present in many Firmicutes, including *Clostridium* species, appears to be coded by gene *ybzG*. Ribosomal protein S12 is thiomethylated at a conserved aspartate (which was a – wrong – asparagine in the published sequence) by protein RimO (YqeV) (Anton *et al.*, 2008).

Several genes code for methylases and acetyltransferases, probably involved in ribosomal protein modification, suggesting that much more work must still be performed on the process of translation in *B. subtilis*. Ribosome assembly is also directed by a variety of energy-dependent proteins such as the YsxC GTPase. Several ATP- or GTP-dependent enzymes may be involved in the process, especially during temperature shifts.

The process of translation also involves proper folding of nascent proteins (Tig and PpiB prolyl isomerases play an important role in the process) as well as degradation of incomplete or chemically altered proteins. We note that we did not find a counterpart for the system repairing isoaspartate in other organisms, suggesting either that its counterpart belongs to the proteins of still unidentified function, or that there is an efficient degradation pathway recognizing isoaspartate (Danchin, 2008).

### Anabolism and salvage

#### Coenzymes.

*B. subtilis* synthesizes all major co-enzymes or prosthetic groups found in free-living bacteria except for coenzyme B12. We shall only stress here features that are original to this bacterium or very recently characterized.

The synthesis of biotin in *B. subtilis* is somewhat unusual. It uses a transaminase with lysine as the amino-group donor, not *S*-adenosylmethionine (AdoMet) as in reference pathways (Van Arsdell *et al.*, 2005).

The metabolism of pyridoxal phosphate is original in *B. subtilis*, as part of the pathway is not similar to that in Bacteria such as *E. coli*, but is rather highly similar to that found in plants and fungi (Raschle *et al.*, 2005).

The general metabolism of thiamin has not been completely unravelled. An alternative pathway to the standard route exists in *E. coli*, but it is not understood. Begley and co-workers have shown that in addition to *de novo* synthesis many salvage pathways exist to scavenge thiamin precursors or derivatives from the environment. The TenA–TenI system associated with regulating the production of extracellular proteases is in fact a widespread salvage system (Begley *et al.*, 2008; Jenkins *et al.*, 2008).

Most of the steps involved in menaquinone/ubiquinone biosynthesis can be identified in the sequence of the genome. However, the counterpart of *ubiG* is not easily uncovered among the possible genes of the pathway. Gene *yrrT*, located upstream of *mtnN* and genes involved in scavenging homocysteine (Andre *et al.*, 2008) could code for the corresponding function yielding *S*-adenosylhomocysteine, but more work needs to be performed to challenge this hypothesis (A. Sekowska, unpublished observations).

#### Carbon metabolism.

*B. subtilis* displays a textbook organization of its carbon metabolism (for reviews see Sauer & Eikmanns, 2005; Sonenshein, 2007). The phenomenon of catabolite repression has been studied in much detail (for recent references see Singh *et al.*, 2008). While the main catabolite repressor CcpA has been identified, the process is far from being completely unravelled (the function of most genes conserved in synteny with *crh* is not understood yet). We hope that the tracks suggested by our annotation will help further discoveries in the domain.

We must also note that some genes involved in carbon metabolism are essential for unexpected reasons. This is the case for *eno*, *fbaA*, *pgm*, *tkt* and *tpi* in the main glycolytic pathway and *odhAB*, coding for ketoglutarate dehydrogenase, for example. Interestingly, analysis of co-evolution of RNase genes suggests the existence of a degradosome in *B. subtilis*, which would be functionally linked to the main glycolytic pathway (co-evolution with *tpi* and *eno* in particular) (Danchin, 2009a).

#### Nitrogen metabolism.

The best nitrogen source for *B. subtilis* is glutamine. We have observed that bacteria grown in minimal medium with ammonium as a nitrogen source grow slowly and evolve rapidly to fast growers (Sekowska, 1999 and unpublished observations). This fits with the demonstration by Belitsky & Sonenshein (1998) that strain 168 can adapt to rapid growth on ammonium or glutamate by a reversible spontaneous duplication or deletion of a 9 bp sequence in the alternative glutamate dehydrogenase gene, *gudB*. This feature may have had significant consequences in the sequencing project by creating unwanted polymorphism, as some members of the consortium were familiar with growth on ammonium, unaware of the possibility that this original phase variation might have consequences in terms of mutation selection. Nitrate can also be used as a nitrogen source, as *B. subtilis* has both a respiratory and an assimilatory nitrate reductase (Nakano & Zuber, 1998). Several salvage pathways for purines and pyrimidines (Christiansen *et al.*, 1997; Tozzi *et al.*, 2006), including salvage of energy-rich nucleotides (Danchin, 2009a), exist in the organism. Adenine deaminase AdeC has activity demonstrated in the purine salvage pathway. The *yerA* paralogue could be a missing dihydropyrimidinase. It is also required for scavenging derivatives of AdoMet. There is another link between nitrogen (via arginine and polyamines) and sulfur metabolism (Sekowska *et al.*, 2001). Further, cysteine protects ArgG against reactive oxygen species (ROS) (Hochgrafe *et al.*, 2007), thus leading to arginine derepression under conditions of excess cysteine or ROS production.

In diaminopimelate synthesis, there is a requirement for the conversion of *N*-acetyl-2,6-diaminopimelate to acetamido-6-oxoheptanoate. Since this reaction belongs to non-essential reactions in *B. subtilis*, there are probably several genes that encode proteins showing this enzymic activity. Transaminase PatA could be a candidate for this activity, as it is located in a region coding for activities involved in chemotaxis, sporulation and control of cell envelope synthesis. Unfortunately, the biochemical data collected on this enzyme only show that it is not involved in methionine transamination (Berger *et al.*, 2003). Other enzymes such as SpsC or NtdA might also display some of the missing activity. Particular attention should be paid to *N*-acetylornithine aminotransferase (EC 2.6.1.11), encoded by gene *argD*. This gene might need better characterization of its enzyme activity, as it might display two related functions, involving both the metabolism of arginine and the metabolism of diaminopimelic acid. Indeed, in *E. coli,* ArgD has both functions despite the fact that the diaminopimelate substrate is succinylated, not acetylated. This would make the *B. subtilis* enzyme particularly prone to have both activities. This might account for the particular feature of the transcription of this gene observed in transcriptome experiments (Sekowska *et al.*, 2001). Biochemical work is needed to substantiate this point.

#### Sulfur metabolism.

Specificities of sulfur metabolism as established from the *B. subtilis* sequence have been described in a review article (Sekowska *et al.*, 2000). However, with the new complete annotation of the genome, novel features emerge that are worth describing here. While synthesis of cysteine and methionine is fairly standard for a Gram-positive organism, the reverse transsulfuration pathway, allowing growth on methionine, is unusual and not completely understood yet (Hullo *et al.*, 2007). Methionine salvage results from recycling of catabolites of AdoMet and from recycling of the start of proteins where the second residue is small, via two methionine aminopeptidases, MapA and MapB (You *et al.*, 2005). AdoMet is mainly used to transfer methyl groups (52 methyltransferases) and results in the production of *S*-adenosylhomocysteine, which is further metabolized by MtnN into *S*-ribosylhomocysteine and adenine. AdoMet is used as the precursor of polyamine after decarboxylation, yielding methylthioadenosine, which is recycled via methylthioribose (MTR) by a complete methionine salvage pathway where the carbon atoms of methionine derive from the ribose moiety, not from TCA (tricarboxylic acid) cycle intermediates (Sekowska *et al.*, 2004). AdoMet is used to make queuosine, a complex modified base near the anticodon of several tRNAs (gene *yqeE*). It is also used as a radical in several reactions coded by genes *bioB*, *hemN*, *hemZ*, *kamA*, *moaA*, possibly *skfB*, *splB*, possibly *ycnL* for a membrane protein, *yfkA* (fused from *yfkA* and *yfkB* in the 1997 sequence), *yloN*, *ymcB*, *yutB*(*lipA*), possibly *yuzB* and *yydG* (Frey *et al.*, 2008). The resulting product of the reaction, 5′-deoxyadenosine, might perhaps be recycled by the MTR salvage pathway (Sekowska *et al.*, 2004).

Genes involved in formation of iron–sulfur clusters have been predicted (Sekowska *et al.*, 2000) and some of them have been discovered experimentally (Kiley & Beinert, 2003). Analysis of the genome predicts several genes involved in the process of construction of iron–sulfur clusters: *nifS*, *yutI*, *iscU*(*yurV*), *sufC*(*yurY*), and possibly *ygaC* and *yneR*.

### General maintenance and protection

#### Reactive oxygen species, nitric oxide.

*B. subtilis* possesses three superoxide dismutases, including one exported lipoprotein. As described above, sulfur metabolism appears to be important for protection against ROS. A nitric oxide (NO) synthase (Chartier & Couture, 2007) uses YkuN and YkuP flavodoxins for electron transfer (Wang *et al.*, 2007). The NsrR response regulator monitors NO (Nakano *et al.*, 2006), suggesting a specific role of this gas in protection against ROS.

Two methionine sulfoxide reductases, MsrA and MsrB, are involved in oxidized methionine repair in a process that is at least in part regulated by the novel regulator Spx (see below) (You *et al.*, 2008).

#### Temperature.

The *B. subtilis* niche implies considerable fluctuations in temperature. The organism harbours a standard arsenal of cold-shock proteins and RNA helicases. It has also two thymidylate synthases, ThyA and ThyB, permitting it to grow at temperatures as high as 55 °C (Montorsi & Lorenzetti, 1993).

#### Salt.

*B. subtilis* is fairly resistant to desiccation and, in parallel, to fairly high levels of sodium. Some experiments suggested that this was mediated by the DegS–DegU system (Dartois *et al.*, 1998) and the sigma-B regulon (Petersohn *et al.*, 2001). However these systems are very general and might not be specific in the process. A significant part of the corresponding adaptation is mediated by synthesis of glutamate as a precursor of proline and may interfere with formation of iron–sulfur clusters (Hoper *et al.*, 2006). In any event, synthesis and uptake of compatible solutes (such as glycine betaine) permit adaptation to high osmolarity.

### Regulation

Genes do not operate in isolation: RNA polymerase activity is regulated at a global level by sigma factors and anti-sigma factors (for reviews see Gruber & Gross, 2003; Helmann, 1999, 2002; Kazmierczak *et al.*, 2005; Kroos & Yu, 2000; van Schaik & Abee, 2005). In some cases there is a coupling with the ribosome via the Nus factors and specific factors such as the ribosome-associated sigma-54 modulation protein YvyD. Integration and sensing is associated in two-component systems, which have been widely discussed previously (see for example Bisicchia *et al.*, 2007; Joseph *et al.*, 2002; Kobayashi *et al.*, 2001).

Specific identification of the regulators (often small molecules) of putative transcription factors is difficult and in most cases it is still unknown. Progress is slow in this domain; yet the interesting case of regulators with pyridoxal phosphate (PLP) sites (*ycxD*, *gabR*(*ycnF*), *ydeF*, *ydeL*, *ydfD*, *yhdI*, *yisV*) suggests coupling between regulation and enzyme activity. Indeed, there are some situations where a regulator is directly involved in an enzyme activity (e.g. biotin biosynthesis: Chapman-Smith *et al.*, 2001) or associated with it (Tanous *et al.*, 2008).

Several regulators often display a global behaviour. This is the case for the Lrp family coded by genes *azlB*(*yrdG*), *lrpA*, *lrpB*, *lrpC*, *yezC*, *yugG*(*alaR*) and *ywrC*, global regulators CcpA, CodY, TnrA (Sonenshein, 2007) and Spx (Beck *et al.*, 2007; Reyes & Zuber, 2008; You *et al.*, 2008). A paralogue of the latter, MgsR (YqgZ), is a transcriptional regulator of stress and modulates the sigma-B response (Reder *et al.*, 2008).

Cyclic di-GMP is a regulatory molecule involved in many processes controlling collective behaviour in bacteria (Sinha & Sprang, 2006); three proteins (YdaK, YkoW and YtrP) have motifs that suggest synthesis of cyclic di-GMP in *B. subtilis*. They are sometimes associated with sites that suggest a role in sensing environmental cues (in YkoW, an additional PAS domain is found between the MHYT and GGDEF domains, suggesting a role in sensing dioxygen, carbon monoxide or NO); protein YybT is a phosphodiesterase-like protein, which has a modified GGDEF motif, suggesting that it could act as the phosphodiesterase involved in cyclic di-GMP control, and YpfA could also hydrolyse cyclic di-GMP, while YdaN is a regulator that has a site which could bind cyclic di-GMP and relay its gene expression control activity in particular during biofilm synthesis. In short, it seems likely that cyclic di-GMP plays a role in gene expression in *B. subtilis*.

### Occupying a niche

#### The cell's standard phenotypes.

Described earlier as *Vibrio subtilis*, *B. subtilis* was reproducibly identified by Zopf in 1885 as isolated from hay soaked in a small volume of water at 36 °C for 4 h, then filtered and boiled for 1 h. Most often, the process ended with a pellicle formed at the surface of water after incubation for 1 day at 36 °C. It was usually exclusively formed by *B. subtilis* bacteria.

In Bergey's manual, Sneath noted that ‘It is generally not possible to draw any conclusions from the site of isolation of a *Bacillus* strain as to its natural habitat’ (Sneath, 1986). However, repeated isolation from hay indicates that *B. subtilis* is an epiphyte, with the phylloplane (and the rhizoplane as a consequence) as the preferred niche. Identification of many genes in the genome strongly supports this observation, and in particular supports the idea that the surface of leaves is a preferred niche of the organism (Table 2).

Strain 168 is auxotrophic for tryptophan. This character is derived from some of the initial mutagenic events associated with improvement of the organism as a laboratory workhorse. For the same reason it is also lacking surfactin production while restoration of pseudogene *sfp* into a functional frame allows bacteria to produce efficiently this molecule, acting both as an antibiotic and as a surfactant used for swimming and swarming (Julkowska *et al.*, 2005; Kunst *et al.*, 1997; Reuter *et al.*, 1999). Leaves often produce H_2_S to avoid overaccumulation of sulfur: this may be scavenged directly by the bacteria, accounting for a complex sulfur metabolism network, enabling good growth on *S*-methylcysteine, for example. Consistent with the phylloplane as a preferred niche, *B. subtilis* grows best with vigorous aeration and this mistakenly placed it for a long time among the obligate aerobes (Nakano & Zuber, 1998). It has for this reason an efficient arsenal of genes combating the effects of dioxygen (including many dioxygenases). Interestingly, it is light sensitive, with a sensor, YtvA (Gaidenko *et al.*, 2006), involved in regulation of gene expression. Finally it has a considerable amount of genes involved in plant maceration, and it is able to swim, swarm and make complex biofilms, which is consistent with strong association with plant leaves.

#### Antibiotics and quorum sensing.

As noticed previously, *B. subtilis* synthesizes a variety of complex molecules via the non-ribosomal peptide synthesis pathway as well as via maturation of peptides (Kunst *et al.*, 1997). These molecules play the role of antibiotics (and this probably accounts for the pure cultures isolated from hay infusions), and are also used as surfactants, permitting smooth development on planar surfaces. While the quorum-sensing AI-2 pathway appears to exist, many other pathways, using short peptides as signals, are coded in the genome. This is consistent with the complex environment of the plant, which produces oxygen in the light of the day time, then CO_2_ during the night, with a concomitant considerable change in temperature and humidity. This is further made more complex by the alternation of seasons, with decay of leaves followed by burgeoning and maturation. Among the processes permitting adaptation to changing conditions is sporulation (see below), but there may also exist some specific chemical adaptations such as synthesis of hopanoids, which would permit resistance to desiccation: YhfL is similar to a squalene hopene cyclase, present in *Thermus thermophilus*.

All these plant-related features require specific adaptation processes, which must work more or less orthogonally to each other. This implies original regulatory setups, marking the gene setup of *B. subtilis* as a goldmine for the construction of novel synthetic biology devices (de Lorenzo & Danchin, 2008).

#### The *B. subtilis* differentiation programmes.

*B. subtilis* has been chosen as a model organism for its original differentiation programme, making spores, which was proposed as representative of the rules controlling differentiation in general (Aguilar *et al.*, 2007). Indeed the study of sporulation has involved thousands of scientists all over the world. This heavily trodden area therefore does not need to be documented further here except to stress the concept of cannibalism, which has recently been emphasized in the context of the way bacterial colonies may behave collectively (Claverys & Havarstein, 2007; Nandy *et al.*, 2007; Ellermeier *et al.*, 2006).

#### Competence.

The discovery of genetic transformation by Avery and his colleagues placed the process of transformation of DNA into cells at the core of the early efforts to construct experimental processes based on the use of the DNA molecule as a genetic tool. This drove the isolation of a *B. subtilis* strain amenable to easy transformation. Gamma-ray mutagenized derivatives of the Marburg strain of *B. subtilis* at Yale were brought to Western Reserve University by Yanofsky, where Spizizen tried a number of strains for efficient transformation and settled on a tryptophan auxotroph of strain 168 (Anagnostopoulos & Spizizen, 1961). Competence was studied by several groups in detail (449 references at PubMed on the topic), and the new annotation of the genome now reveals novel features such as requirement for LipA (YutB), an AdoMet radical enzyme, for establishment of competence (Ogura & Tanaka, 2009), or further details of the DNA uptake machinery, such as involvement of DprA (Smf) (Tadesse & Graumann, 2007).

#### Sporulation.

The process of sporulation is the best-documented behaviour of *B. subtilis*. As a matter of fact this organism has been used as a model for cell differentiation, with the study of sporulation as the paradigm. The keyword ‘subtilis’ is associated with 768 articles with keyword ‘spor*’ in PubMed at the time of writing, with 21 review articles since 1993. One observation may be worth mentioning: the analysis of the fine structure of the proteome using correspondence analysis suggests that some proteins involved in the process of sporulation are related to proteins performing phage functions. This is in line with the observation that the SinR repressor, a key regulator in sporulation, has a domain that is similar in tertiary structure to that of a lambdoid repressor (Lewis *et al.*, 1998). A thorough phylogenetic study should explore the conjecture that some phage functions might have been recruited at the origin of sporulation.

#### Swimming, swarming and forming biofilms.

More recently, the collective behaviour of *B. subtilis* has been analysed in terms of other differentiation processes such as those permitting swimming, swarming and forming biofilms (see Table 2) and the literature is growing fast in the domain, in particular with systems biology approaches (Rajagopala *et al.*, 2007). While cellulose is now well understood as a core component of biofilm structures, the role and synthesis of polyglutamate has more recently been emphasized, including for industrial applications (Meerak *et al.*, 2008).

### Horizontal gene transfer

As discussed above, *B. subtilis* harbours many genomic islands which display a variety of specific features in terms of DNA composition, codon usage biases and alteration of syntenies. Several are the hallmark of horizontal gene transfer, most often via integration of prophages, which can either remain functional (such as SP*β*), retain some activity (PBSX, *skin*), or are in the process of genetic decay (Kunst *et al.*, 1997). Associated with these genomic islands we find a variety of genes that keep signatures of widespread functions permitting gene transfer. *whiA* (*yvcL*) is a distant homologue of LAGLIDADG homing endonucleases that retained only DNA binding (Knizewski & Ginalski, 2007). It is present in Gram-positive bacteria (both A+T- and G+C-rich) and present in mycoplasmas, suggesting that some sort of retrotranscription existed very early on. Other features, such as those displayed by genes *tilS* (lysidine synthesis), *smc* (chromosome segregation) *divIVA*, *sepF*(*ylmF*) and others, are related to genes present in Eukarya. This is consistent either with a common ancestry of the corresponding functions at some point in evolution, or with the ‘phagocytosis’ scenario of the origin of living cells where phagocytic eukaryotes acting as predators would have predated the appearance of Bacteria, endowed with complex envelopes permitting them to escape predation, or Archaea, which would have fled to harsh niches where they could not be reached by predators (Kurland *et al.*, 2007).

### Sequence and annotations for all

As a result of the functional reannotation presented above, gene products and gene names used in SubtiList were significantly revised. In particular, 407 genes whose previous name started with the letter ‘y’ (meaning that their function was unknown) were given a biologically significant name, on the basis of experimental evidence found in the literature. In addition, over 3000 new bibliographical references were imported in the database and linked to the relevant genes.

The new sequence and annotation information is integrated in an updated version of SubtiList (Moszer *et al.*, 2002). This genome database is now part of a multi-genome framework, GenoList – http://genolist.pasteur.fr/GenoList (Lechat *et al.*, 2008) – which holds genome information of more than 700 prokaryotic organisms imported from the Genome Reviews repository (Sterk *et al.*, 2006), and replaced in a few cases by manually curated genome data, such as those described in this work (accessible at http://genolist.pasteur.fr/GenoList/Bacillus subtilis 168). GenoList enables comparative genome analysis together with browsing and query capabilities similar to those of the previous version of SubtiList (Moszer *et al.*, 2002). In addition, metabolic pathway reconstruction data are available in a BioCyc-type web server, SubtiliCyc (http://genocyc.pasteur.fr), which is dynamically linked to GenoList. The sequence and annotation is also available on the MaGe platform (http://www.genoscope.cns.fr/agc/mage) as project BacilluScope (Vallenet *et al.*, 2006).

The EMBL accession number for the sequence reported in this paper is AL009126.

### Conclusion

Model bacteria play an essential role in defining reference knowledge, after which a considerable fraction of the knowledge on bacteria is accumulated. *Bacillus subtilis* 168 is the reference organism for the Firmicutes and it is therefore essential to have a particularly accurate sequence of the organism, with up-to-date annotations. In the PubMed reference library 23 000 articles refer to some aspect of *B. subtilis* biology, while one finds 2 million pages at Google and 208 000 at Google Scholar, showing that this bacterium indeed plays the role of a model bacterium. We hope that the present effort in resequencing and reannotating the genome will benefit the international community of microbiologists. In the annotation process we used as much as possible the full content of the articles present in data libraries (Open Access publications and publications at PubMed Central). This permitted us to propose a few educated guesses about gene functions that will need to be experimentally validated. A general updated metabolic schema of the organism, SubtiliCyc, is available via the GenoList environment. Among the interesting metabolic features of the organism are many pathways directly associated with interaction with plants. As an example of the guesses we propose to the reader, triggered by our interest in sulfur metabolism (Sekowska *et al.*, 2000), is the prediction of YoaDC and YoaE as being involved in cysteine degradation, with transfer of sulfur to the C-terminal end of YoaD producing glycerate, which is subsequently modified to phosphoglycerate by YoaC; YoaD could be an important sulfur donor for construction of Fe–S clusters or sulfur-containing coenzymes. Using cysteine directly, it could be used for cyanide detoxification in plants. Many other examples of this type can be found in the present annotation of the genome sequence. We hope this will act as an incentive to trigger further work to substantiate these inferences.

## Figures and Tables

**Table 1. t1:** Comparing the new sequence with the old one

**Gene comparisons between the old and the new version of the annotations**	No. (per cent) of genes*
Identical genes	3323 (78.3 %)
Amino acid variations	426 (10.0 %)
Adjusted start codons	50 (1.2 %)
C-terminal variations only	221 (5.2 %)
N-terminal variations only	4 (0.09 %)
C-terminal and N-terminal variations	11 (0.26 %)
Fusions	20 (0.47 %)
Fissions	20 (0.47 %)
Newly annotated genes	171† (4.0 %)

*In the case of fusion/fission events, the number of new genes resulting from the event is indicated. †Including 48 pseudogenes or gene remnants.

**Table 2. t2:** Genes of the cenome suggesting that *B. subtilis* is an epiphyte

**Response to dioxygen**	**Response to light**	**Maceration of leaves**	**Unusual sulfur metabolism**	**Plant-related genes**	**Swimming, swarming and miscellanea**
*spx*	*ytvA*	*yesLMNOPQRS*	*ytlI ytmI tcyJKLMN ytmO ytnIJ rbfK ytnLM*	*pdxS pdxT*	Locus *tnrE* locus *sfr*
*perR*		*rhgT yesUVWXYZ*	*yxeIJKLMNOPQ*	*pyrD pyrK*	*swrAA swrAB swrB yabR*
*ypoP msrAB*		*yetA lplABCD*	CymR and CysL regulons	*thrB thrC*	*aprE bpr mpr nprE vpr wprA*
*hemAT mtnD*		*pelC yoaJ*		*mtnW*	*yhfL salA sinIR tasA*
*nosA*		*xsa lacA xynA xynD yvfM araABDLMNPQ abfA araE yvfO araA abnA yxiA*			*ydaM yfiQ ykfABCD ylbF ymcA yoaW ypfA yqhH yqxM yulF ywqH yxaM yxjH yydFGHIJ*
*ydfO yetH yodE yrkC yrpB yubC*		*bglC bglH bglS*			*epsABCDEFGHIKLMNO*
*catDE cdoA mhqA qodI*		*yoaJ (exlX)*			*ecsB comP*
		Glucomannan utilization operon (*gmuBACDREFG*, formerly *ydhMNOPQRST*)			

## Abbreviations

- AdoMet, *S*-adenosylmethionine
- CDS, coding sequence
- IIMP, integral inner-membrane protein
- MTR, methylthioribose
- RGP, regions of genomic plasticity
- ROS, reactive oxygen species
